# Gravity Threshold and Dose Response Relationships: Health Benefits Using a Short Arm Human Centrifuge

**DOI:** 10.3389/fphys.2021.644661

**Published:** 2021-05-11

**Authors:** Chrysoula Kourtidou-Papadeli, Christos A. Frantzidis, Sotiria Gilou, Christina E. Plomariti, Christiane M. Nday, Dimitrios Karnaras, Lefteris Bakas, Panagiotis D. Bamidis, Joan Vernikos

**Affiliations:** ^1^Biomedical Engineering & Aerospace Neuroscience, Laboratory of Medical Physics, Faculty of Health Sciences, School of Medicine, Aristotle University of Thessaloniki, Thessaloniki, Greece; ^2^Greek Aerospace Medical Association and Space Research, Thessaloniki, Greece; ^3^Aeromedical Center of Thessaloniki, Thessaloniki, Greece; ^4^Laboratory of Aerospace and Rehabilitation Applications “Joan Vernikos” Arogi Rehabilitation Center, Thessaloniki, Greece; ^5^Thirdage llc, Culpeper, VA, United States

**Keywords:** artificial gravity, blood gas analysis, cardiac output, deconditioning, heart rate variability, mean arterial pressure, short-arm human centrifuge

## Abstract

**Purpose:**

Increasing the level of gravity passively on a centrifuge, should be equal to or even more beneficial not only to astronauts living in a microgravity environment but also to patients confined to bed. Gravity therapy (GT) may have beneficial effects on numerous conditions, such as immobility due to neuromuscular disorders, balance disorders, stroke, sports injuries. However, the appropriate configuration for administering the Gz load remains to be determined.

**Methods:**

To address these issues, we studied graded G-loads from 0.5 to 2.0g in 24 young healthy, male and female participants, trained on a short arm human centrifuge (SAHC) combined with mild activity exercise within 40–59% MHR, provided by an onboard bicycle ergometer. Hemodynamic parameters, as cardiac output (CO), stroke volume (SV), mean arterial pressure (MAP), systolic blood pressure (SBP), diastolic blood pressure (DBP), and heart rate (HR) were analyzed, as well as blood gas analysis. A one-way repeated measures ANOVA and pairwise comparisons were conducted with a level of significance *p* < 0.05.

**Results:**

Significant changes in heart rate variability (HRV) and its spectral components (Class, Fmax, and VHF) were found in all g loads when compared to standing (*p* < 0.001), except in 1.7 and 2.0g. There were significant changes in CO, cardiac index (CI), and cardiac power (CP) (*p* < 0.001), and in MAP (*p* = 0.003) at different artificial gravity (AG) levels. Dose-response curves were determined based on statistically significant changes in cardiovascular parameters, as well as in identifying the optimal G level for training, as well as the optimal G level for training. There were statistically significant gender differences in Cardiac Output/CO (*p* = 0.002) and Cardiac Power/CP (*p* = 0.016) during the AG training as compared to standing. More specifically, these cardiovascular parameters were significantly higher for male than female participants. Also, there was a statistically significant (*p* = 0.022) gender by experimental condition interaction, since the high-frequency parameter of the heart rate variability was attenuated during AG training as compared to standing but only for the female participants (*p* = 0.004).

**Conclusion:**

The comprehensive cardiovascular evaluation of the response to a range of graded AG loads, as compared to standing, in male and female subjects provides the dose-response framework that enables us to explore and validate the usefulness of the centrifuge as a medical device. It further allows its use in precisely selecting personalized gravity therapy (GT) as needed for treatment or rehabilitation of individuals confined to bed.

## Introduction

Life evolved and adapted within the gravitational field of Earth and gravity is critical to our existence, since we have to rely on Earth’s gravity, as a fundamental reference, in structuring musculoskeletal support and in the organizing of bodily fluids. Along the years, we evolved specialized motion-sensing receptors in our inner ears acting like biological guidance systems. Also, the cardiovascular system has evolved to operate in Earth’s gravity while standing, sitting or lying down. Years of space research in weightlessness and analog environments led to the conclusion that physiological health and performance on Earth is significantly dependent on gravity (Vernikos et al., 1996; Vernikos, 1996, 1997, 2008, 2017; Blaber et al., 2013b; Tanaka et al., 2016). When gravity is not used efficiently (due to lifestyle, inactivity or bedrest) to maintain the level of health that is appropriate to living in Earth’s 1G environment, it leads to negative consequences in all human physiological systems (Sandler and Vernikos, 1986; Rittweger and Frings-Meuthen, 2013; McDonnell et al., 2019). Orthostatic intolerance (OI), an inability to regulate blood pressure (BP) on assuming an upright stance (Robertson, 1999; Lambert and Lambert, 2014), is commonly experienced by astronauts upon their return to Earth after both short and long duration spaceflight (Buckey et al., 1996; Vernikos, 1996; Lee et al., 2015), as well as in people who resume posture after being confined to bed for experimental, clinical or pathological reasons (Goswami, 2017; Goswami et al., 2017).

We now know from Space research, how human physiology is influenced by the lack of gravity loads (Gz) either acutely or over extended periods of time, with detrimental consequences on all organ systems of the body (Goswami, 2017; Goswami et al., 2017). Sitting and inactivity on Earth are also associated with an elevated risk of mortality from all causes, including cardiovascular disease (Katzmarzyk et al., 2009).

In the upright posture, gravity acts along the head to feet column and pulls fluids from the head to the feet along the Gz axis, leading to a BP decrease at brain and heart level and a significant increase at foot level (Furst, 2020). Removal of hydrostatic gradients, occurring in microgravity conditions and analog environments like bedrest and immobilization, or in physically impaired individuals, triggers adaptations in human physiology to compensate for these changes (Vernikos et al., 1996; Rittweger and Frings-Meuthen, 2013; McDonnell et al., 2019). In the supine position, gravity is applied across the short, chest-to-back fluid column (Gx) with equal BP at the levels of brain, heart and feet.

Standing up after bedrest, challenges the cardiovascular system due to the changes in fluid pressure and redistribution of fluid volume to the feet, resulting in involuntary reflex adjustments needed to get the blood back up to the head (Convertino et al., 1990). Such studies are of fundamental importance to physiologists, helping them understand how the human body adapts to both microgravity and bed rest as well as identify the underlying mechanisms involved in standing up after bedrest without fainting.

Space physiologists initially focused on adaptation processes to develop strategies (countermeasures) to mitigate the detrimental effects in human physiology resulting from the lack of gravity in space. Countermeasures tested included nutrition, vibration, or electrical stimulation. Though artificial gravity (AG) was also proposed, it was never tested systematically in space due to logistical and cost issues. (Goswami et al., 2008; Blaber et al., 2013a; Hargens et al., 2013). Studies using bedrest (BR) or head down tilt bedrest (HDTBR) on the ground to induce microgravity-related changes (LeBlanc et al., 1988; Jeong et al., 2013; Norsk, 2014) also indicated that these countermeasures were only partially effective. It should be noted that these countermeasures were administered once a day (Evans et al., 2004; Stenger et al., 2007, 2012; Hargens et al., 2013). However, when administered multiple times throughout the day, either standing alone or upright exercise, proved to be the most effective countermeasure (Vernikos et al., 1996). Based on these observations, the intermittent administration of Gz with or without exercise using a short-arm human centrifuge (SAHC) was proposed as the most promising countermeasure for mitigating the physiological multisystem deconditioning in space or bed rest (Vernikos, 1997; Evans et al., 2004; Frett et al., 2014; Clément et al., 2015).

The absence of hydrostatic pressure in 0g conditions, causes fluid shifts among compliance vessels and changes in the cardiovascular system, in flow resistance and in diastolic cardiac performance (Peterson et al., 2002), eliciting compensatory neurohormonal responses, such as those from carotid and atrial mechanoreceptors, vascular smooth muscle, stretch receptors, and several hormones. To counteract the effect of gravity on Earth, humans have developed neuronal and humoral adjustments in order to maintain a steady mean arterial pressure (MAP) in case of sudden change in posture or sudden acceleration. We considered that increasing acceleration and Gz while supine to mimic standing conditions would be beneficial not only to astronauts in space but also to bedrest patients or patients with immobility due to pathological causes such as neuromuscular diseases, osteoporosis, OI, geriatric individuals, and vestibular disorders. Daily posture change such as intermittent standing has been shown to counteract short term deconditioning (Convertino et al., 1990; Duvivier et al., 2018). There is a system-specific benefit of daily standing up in Earth’s 1G and there is a preventive value of exposure to daily short-period passive +1Gz vertical acceleration, or activity in +1Gz (Vernikos et al., 1996). Intermittent AG may effectively “train” cardiovascular or neuromotor mechanisms responsible for maintaining orthostatic integrity. However, the appropriate configuration including optimal g-level, angular velocity, gravity gradient, and exercise protocol have not been systematically established.

Goswami et al. (2015) used two G loads, – 1 or 2g at foot level – and monitored continuous BP and heart rate (HR). They concluded that the response of the cardiovascular and cerebrovascular systems during centrifugation with 2-g at the feet, was analogous to the orthostatic challenge exerted by standing up in natural gravity (1Gz).

In order to evaluate optimal G load, compared to standing, the present study established dose-response curves of significant cardiovascular indices. Hemodynamic parameters along the sequential levels of 0.5, 0.7, 1, 1.2, 1.5, 1.7, and 2.0g were integrated to construct dose response curves in order to identify the optimal G load that would be equivalent to standing and beneficial for rehabilitation and training. We simultaneously recorded non-invasively cardiac output (CO), stroke volume (SV), MAP, systolic blood pressure (SBP), diastolic blood pressure (DBP) and heart rate (HR), as well as blood gas analysis measurements of partial pressure of oxygen (pO_2_), partial pressure of carbon dioxide (pCO_2_), oxygen content (O*^–^*), carbon dioxide content (CO_2_^–^) and oxygen saturation (O_2_sat%). We specifically selected CO, SV, and MAP, since studies of extended bed rest in humans have revealed significant reduction in cardiac SV and CO when activity and posture change were restricted (Saltin et al., 1968). This data is designed to serve as a guide in identifying AG rehabilitation regimes for those following inactivity or bedrest, various mobility disorders or to mitigate detrimental effects of microgravity in astronauts.

This study proposes a robust analysis framework. More specifically, it includes a variety of G loads (seven separate G loads) as well as a large number of outcome measures. The combination of these two novelty factors will facilitate the use of AG over a range of pathologically induced immobility conditions.

## Materials and Methods

### Participants

Twenty-eight healthy volunteers (15 males and 13 females), aged 20–45 years. were recruited by the Aeromedical Center of Thessaloniki, Greece, to participate in this study to evaluate the physiological responses to AG. All participants were physically active, healthy, non-smokers, with no medical history of chronic disease. None of the participants had any history of cardiovascular, metabolic, or neurological diseases, and None of them were professional or elite athletes. Female participants had to test negative on a pregnancy test before enrollment in the study. There were non-physiological cardiovascular features for two participants and missing values for another two participants. So, they excluded from further analysis, resulting thus in twenty-four participants. Their demographics characteristics are reported in Table 1.

**TABLE 1:** Participants’ demographics for gender, age, height, weight, and BMI.

Demographics	Classes	Frequency	Percentage (%)
Gender	Male	12	50
	Female	12	50
	Total	24	100
Age	20–32	20	83.3
	33–45	3	12.5
	46+	1	4.2
	Total	24	100
Height	1.57–1.66	7	29.2
	1.67–1.76	10	41.7
	1.77–1.86	4	16.6
	1.87–1.96	2	8.3
	1.97+	1	4.2
	Total	24	100
Weight	49–60	5	20.8
	61–72	7	29.2
	73–84	7	29.2
	85–96	2	8.3
	97–108	3	12.5
	Total	24	100
BMI	Underweight (<18.5)	1	4.2
	Normal (18.5–25)	15	62.5
	Overweight (25–30)	6	25
	Obese (30–35)	2	8.3
	Total	24	100

**FIGURE 1:**
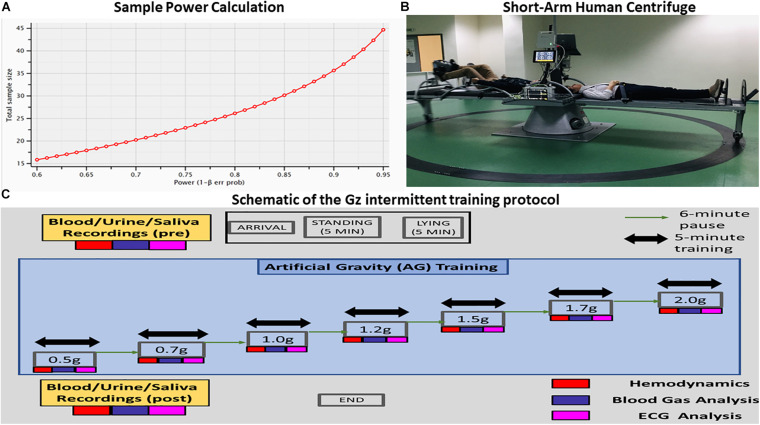
**(A)** Visualization of the analysis performed for estimating the proper sample size for the proposed statistical analysis. The power analysis indicated that the proper sample size differed between parametric (28) and nonparametric tests (27). **(B)** Visualization of the short-arm human centrifuge with two rotating beds. Each bed is equipped with either aerobic or resistance training. There is also the feasibility of artificial gravity (AG) training with no exercise at all in the case of patients suffering from severe mobility difficulties. Cardiovascular biomarkers are monitored in real time, providing thus indices of the exercise intensity and alerts in case of critical situations. Multi-modal neurophysiological analysis during the exercise is being performed through a portable Nihon Kohden EEG device with 32 electrodes mapping electroencephalography (EEG), electrocardiography (ECG), electromyography (EMG) and electro-oculogrammic (EOG) activity. **(C)** Schematic of the +Gz intermittent training protocol. Each phase is labeled accordingly, indicating the corresponding +Gz load over time in minutes.

Exposure to whole body centrifugation is demanding and the number of enrolled participants is often limited. Therefore, we used a power calculation using G-Power 3.1 (Faul et al., 2013) for an error probability (α) of 0.05, a power (1 − β) of 0.80 and an average effect size (*d*) of 0.5 (Figure 1A). In order to achieve statistical significance for AG levels, this analysis yielded an estimated total sample size of 28 participants in the case of non-parametric analysis (Wilcoxon signed-rank test) and 27 for parametric testing (paired *t*-test). Our study finally enrolled 24 healthy participants (12 females). This population is compatible with previous studies in which cognitive function and hemodynamic parameters were assessed during varying gravitational loading (Stenger et al., 2007; Duda et al., 2012; Masatli et al., 2018; Winter et al., 2018; White et al., 2019; Laing et al., 2020) (*N* = 12–28).

### Participant Selection Criteria

Inclusion criteria for participation were the following: 18–45 years old, absence of neurological or psychiatric disorder, absence of vertigo, nausea or chronic pain, no history of syncope, adequate level of physical fitness for the ability to work out aerobically for half an hour. Participants with a height greater than 2 m, elite athletes, with chronic use of substances (drugs or alcohol), with recent (within 6 months) surgery, current arrhythmias, severe migraines, pregnancy, epilepsy, cholelithiasis or kidney stones, dehydration, recent wounds from surgery, recent fractures, acute inflammation or pain, newly inserted metal pins or plates and newly implanted stents were excluded from the study.

After extensive baseline physiological data collection and passing a comprehensive medical examination, they joined the Laboratory of Aerospace Applications and Rehabilitation “Joan Vernikos” at AROGI Rehabilitation Center that houses the SAHC, where the study was conducted.

Participants were fully briefed about the protocol, the sensations they might experience and possible adverse consequences of the acceleration (nausea, dizziness, etc.). Prior to the first study day, each participant was again familiarized with all aspects of the study, including the equipment and personnel involved. They underwent an orientation session on the centrifuge before the start of the study.

At any time, participants had the option to press a “panic button” if they wished to abort the test. Direct verbal contact was maintained between the study physician and each participant. All participants gave their written consent after being informed of any potential risks. They were also asked to complete participation questionnaires for eligibility criteria. The written consent forms were securely stored at the Aeromedical Center. The study was reviewed and approved by the Bioethical Committee of the School of Medicine of the Aristotle University of Thessaloniki (179/19.03.2020).

### Study Protocol

An intermittent centrifugation protocol was selected to assess cardiovascular responses including maintenance of baroreflex and parasympathetic activity (Clément and Bukley, 2007; Clément et al., 2015). The study protocol was registered in the ClinicalTrials.gov (Identifier: NCT04369976).

Participants were advised to abstain from caffeine during the test day, avoid alcohol, and any medications during the preceding 12 h, avoid eating 2 h before the test and avoid exercising heavily 24 h before and after the test.

Participants joined the lab at a prearranged time, approximately the same time every study day – around noon for all – and biological material (blood, urine, and saliva) was taken before and after the end of each study. The participants were connected to non-invasive monitors. Clear Sight monitor^1^ for hemodynamic measurements (CO, MAP, SV, SBP, DBP, and HR), Cnoga Tensor Tip MTX for blood gas analysis (pO_2_, pCO_2_, PH, O ^–^, CO2^–^, and O_2_sat%) and Nihon device for ECG and HRV measurements in different body positions such as standing, supine, 0.5, 0.7, 1, 1.2, 1.5, 1.7, and 2g (Figure 1C – schematic of the Gz intermittent training protocol).

We combined centrifugation on the SAHC with mild intensity exercise based on % maximum heart rate/maximum heart rate (MHR), due to recent studies suggesting that intermittent centrifugation combined with exercise has greater beneficial effects on the cardiovascular system, avoiding OI, and decreasing the possibility of pre-syncope during acceleration due to blood flow rushing towards the feet and contracting calf muscles to increase venous return (Yang et al., 2007; Bonjour et al., 2010; Yang et al., 2010; Diaz-Artiles et al., 2018). The symptoms considered as OI included nausea, sweating, confusion, dizziness, a narrowing of the visual field or the presence of hemodynamic decompensation criteria, such as new-onset ECG abnormalities, an abrupt drop in MAP of >20 mm Hg or a critical narrowing of the pulse pressure.

We adhered to international standards to ensure the safe installation and operation of the device in accordance with the international Directive 2006/42 EC and National Directive FEK 97/A/25-06-2010. Declaration of conformity (CE) marking was affixed in 2019, showing compliance with all relevant Directives and the Essential Health and Safety Requirements (EHSR) detailed within them. Additionally, the device was approved by the Calibration and Quality Services in 2020 (CQS) with calibration certificate number 399 × 0920. The construction of the device was funded by the Aeromedical Center of Thessaloniki, Greece. A safety harness was placed on the participants’ body and they were strapped with their head close to the center of the 2 m-radius centrifuge.

All signals were verified for quality and integrity, and safety procedures were reviewed. The participants’ eyes were covered with an eye cover and lights were off to remove visual cues during rotation. They were asked to avoid moving their heads during centrifugation to avoid Coriolis effect.

Participants remained in the standing position for 5 min. They were then asked to lie down on the centrifuge for 5 min for baseline data collection. They were exposed for a total of 35-min centrifugation intermittently administered for 5 min at each g load, followed by a 6 min “wash-out period” (pause). Gz included 0.5g (approximately 16 rpm), 0.7g (approximately 19 rpm), 1g (approximately 23 rpm), 1.2g (approximately 25 rpm), 1.5g (approximately 28 rpm), 1.7g (approximately 30 rpm), and 2.0g (approximately 32 rpm). The rpms were adjusted according to the participant’s height. The participants spun relatively fast and then stayed at the desired G level for 5 min. During each G load the mean value of the last 60 s was considered for evaluation. There were 7 × 5-min sessions of centrifugation and 7 × 6-min wash out periods. The total time of the procedure, including 5 min sitting and 5 min lying down and time to fasten seat belts, was 1 h and 40 min. After the 1.7 and 2.0g phase, there was a gradual reduction back to +1Gz and to level zero Gz.

For the hemodynamic measurements and monitoring, non-invasive medical devices were used. The Clear Sight beat to beat non-invasive cardiovascular monitor was attached to each participant’s finger for continuous measurements. The participants were asked to hold their hand at heart level and not move them for the entire centrifugation protocol due to the height reference that those devices have, since they are supposed to be connected at heart level to counteract of hydrostatic changes that occur during centrifugation. If signs of OI were present, the +Gz level was reduced or the protocol was terminated. Fortunately, no such phenomenon occurred and all participants were able to successfully complete the experimental protocol. Simultaneously, another noninvasive device, a Cnoga Tensor Tip MTX device was attached to the subject’s finger for blood gas analysis as well as oxygen saturation measurements.

The purpose of implementing a graded +Gz protocol was to reduce the occurrence of OI due to rapid inductions of high loads. Therefore, the first Gz loads from 0.5 to 1g (mild hyper-gravity) were used to reduce any sudden occurrence of an OI event and to prime the baroreflex system for a moderate gravitational stress stimulus. The g loads of 1.5, 1.7, and 2g induced the desired cardiovascular reactions. The wash-out period allowed for a return to baseline conditions before starting the following centrifugation round.

Systolic blood pressure mmHg, DBP mmHg, MAP mmHg, CO L/min, SV mmHg, and heart rate (HR), as well as partial pressure of oxygen (pO_2_) mmHg, partial pressure of carbon dioxide (pCO_2_) mmHg, oxygen content (O^−−^), carbon dioxide content (CO_2_^−−^), and oxygen saturation (O_2_sat%) were monitored continuously and non-invasively with finger plethysmography as the Clear sight (Edwards Life Sciences), the Cnoga Medical Tensor Tip (MTX device) and the Nihon device for continuous EEG/ECG monitoring, but only ECG is considered here. Total peripheral resistance (TPR) was calculated by dividing MAP/CO. Cardiac power (CP) was calculated as {MAP × CO}/451, where MAP = [(systolic blood pressure - diastolic blood pressure)/3] + diastolic blood pressure. To control for significant baseline anthropometric gender differences, CO, and SV were converted to cardiac index (CI L/min/m^2^) and stroke volume index (SVI ml/m^2^).

Additionally, HRV analysis was performed. The pre-processing for HRV consisted of normalization of the ECG time-series in the range of 0–1 in order to facilitate the robust detection of R peaks. The R peak detection was performed through a custom-based script in MATLAB, which estimated peaks greater than a given threshold (≥0.9), while the minimum distance between two consecutive R peaks was set at 0.6 s. The algorithm was previously validated in a semi-automatic sleep detection algorithm (Chriskos et al., 2018).

For the HRV Analysis, since the R–R timeseries is not uniformly sampled across time and does not share the same sampling frequency as the ECG, traditional methods like the Fourier transform are not robust in describing the signal properties. Therefore, we selected to use the Lomb–Scargle periodogram (LSP) in order to extract spectral HRV features (Ruf, 1999). This analysis resulted in the estimation of the relative energy contribution of the following frequency bands: (a) ultra low frequency (ULF) < 0.0033 Hz, (b) very low frequency (VLF) in the range 0.0033–0.04 Hz, (c) low frequency (LF) in the range 0.04–0.15 Hz, (d) high frequency (HF) in the range 0.15–0.4 Hz, and (e) very high frequency (VHF) > 0.4 Hz. The algorithm also estimates the frequency with the maximum power as well as the class in which it belongs. The latter is a scalar number in the range 1–5 (ULF: 1, VLF: 2, LF: 3, HF: 4, and VHF: 5). We also estimated the ratio LF/HF.

### Statistical Analysis

The last 60 s from each phase were averaged and used for the statistical analysis of the hemodynamic variables, in order to highlight the maximal cardiovascular response (Habazetti et al., 2016), and as steady state without the interference of acute changes in +Gz transitions and with minimum variability (Diaz Artiles et al., 2016).

The goal of the analysis was to record a pattern of each cardiovascular variable in different AG loads and more specifically to check the correlations of those variables among standing body position and different g loads exerted at the feet of the individual.

Statistical tests were performed using SPSS (IBM SPSS Statistics 25). In this analysis, the 8 AG levels (standing_5 min, 0.5, 0.7, 1.0, 1.2, 1.5, 1.7, and 2.0g) were merged to five [standing_5 min, ultra-low intensity (0.5g), low intensity (0.7 and 1.0g), medium intensity (1.2 and 1.5g), and high intensity (1.7 and 2.0g) or to 3 (standing_5 min, 1.7 and 2.0g).

The five AG intensity levels were tested for normality using the Kolmogorov–Smirnov test and for homoscedasticity using a Levene’s test. A one-way repeated-measures ANOVA was conducted with AG levels (5 or 3) as the within-subjects factor on biosignal features which served as the dependent variables. In addition, pairwise comparisons were calculated using the Bonferroni *post hoc* procedure. In all cases, significance was taken at the *a* = 0.05 level. Biosignal features are presented as the mean ± standard deviation (Table 2).

**TABLE 2:** Description of the biomarkers that showed a statistically significant main effect of gender when comparing the AG training levels with the standing condition.

Biomarker	Significance	Descriptive statistics
CO	*F*(1,11) = 16.269, *p* = 0.002	Male (7.559 ± 1.539); female: (5.808 ± 1.0853)
CP	*F*(1,11) = 8.071, *p* = 0.016	Male (685.5 ± 188.8); female: (522.9 ± SD: 179)

When resulting data violated an assumption of one-way repeated-measures ANOVA, a non-parametric Friedman’s ANOVA was used for comparison of AG levels (5 or 3) on bio signal features. Furthermore, non-parametric *post hoc* procedures conducted using Wilcoxon signed-rank tests, but correcting for the number of comparisons (the Bonferroni correction).

For HRV statistical tests were performed using SPSS (IBM SPSS Statistics 25). Spectral properties (Class, Fmax, VLF, LF, HF, VH, and LF/HF) at each AG intensity level (standing_5 min, 0.5, 0.7, 1.0, 1.2, 1.5, 1.7, and 2.0g) were tested for normality using the Kolmogorov–Smirnov test and for homoscedasticity using a Levene’s test. A one-way repeated-measures ANOVA was conducted with AG levels as the within-subjects factor on spectral properties which served as the dependent variables. In addition, pairwise comparisons were calculated using the Bonferroni *post hoc* procedure. In all cases, significance was taken at the *a* = 0.05 level. When resulting data violated an assumption of one-way repeated-measures ANOVA, a non-parametric Friedman’s ANOVA was used for comparison of AG levels on spectral properties. Furthermore, non-parametric *post hoc* procedures conducted using Wilcoxon signed-rank tests, but correcting for the number of comparisons (the Bonferroni correction).

Additionally, correlation analysis was performed and significant correlations were found for MAP and CO. The data collected from the participants in nine time-instances (standing pre, supine, 0.5, 0.7, 1.0, 1.2, 1.5, 1.7, and 2.0g) were checked to determine whether they followed a normal distribution. In some cases, this hypothesis was rejected. Therefore, Spearman correlation was performed in all datasets through the Python 2.7 environment with the use of Pandas library, since it is ideal for the evaluation of monotonic relationships between values, whether they are linear or not (Lehman et al., 2005). The correlation matrix was constructed, where ρ values of relationships with no statistical significance (*p* > 0.05) were set equal to 0. The main diagonal matrix the matrix was set to 0 as well.

### Short Arm Human Centrifuge

The short-arm human centrifuge (SAHC) was proposed as a promising countermeasure and training tool within the context of space exploration (Evans et al., 2004; Frett et al., 2014; Clément et al., 2015). The new constructed SAHC (Patent 1009812/13/09/2019, EP3795131), (Figure 1B) is located at the Laboratory of Aerospace and Rehabilitation Applications “Joan Vernikos” at the premises of AROGI Rehabilitation Center, with a diameter of 4 m, with 4-bed capacity and is powered by electricity with a capability of a maximum +3.5 Gz-load at the feet, equipped with a detachable cycle ergometer and resistance pedals with elastic bands (Figure 1B). It was constructed according to both national and international regulations and appropriate certification for function and safety, suitable for patients with restricted mobility disabilities and the capability of gathering physiological data during centrifugation.

Before the study initiation, the height and weight of each participant were measured and more importantly, so was the distance from the central axis to the feet, in order to calculate the g-load at the feet. The head of the participant was placed close to the center of centrifuge rotation, while the feet were directed outwards from the center of rotation in order to allow linear distribution of the g-load in a head-to-toe axis. The g-load at the feet depended on the distance from the axis and the rotational speed of the centrifuge. The g load at the head level was close to zero. Although this does not simulate the typical hydrostatic difference created by standing up in natural gravity, it does generate similar physical stressors in the direction of the feet. Participants successfully completed activity at a voluntary level based on 40–60% of the MHR, provided by an onboard bicycle Ergometer during centrifugation, which they tolerated well, including during the spin-up and spin-down phases of each run.

### Exercise Activity

Although the activity was at a voluntary level provided by the onboard bicycle ergometer, it was controlled and directed not to exceed or decrease the pace according to their personalized target heart rate zone, calculated based on both % MHR and Karvonen formula corresponding to 40–59% of MHR and 30–49% Karvonen corresponding to 5–8 METS (DeVries and Housh, 1994, p. 297; Wilmore and Costill, 1994, p. 524; Powers and Howley, 1997, p. 292*)*. Karvonen formula was reasonably accurate method for estimating exercise intensity as demonstrated by Davis and Convertino (1975). Both methods seemed to be similar. The subjects were monitored real-time via a Bluetooth Polar H10 device, which is an accurate heart rate sensor, and the intensity of the exercise was kept at light intensity pace. The subject was followed during the whole process and was advised accordingly to keep exercise pace within the predetermined limits. The light intensity exercise was verified by HRV recorded on an ECG device placed on the subject during centrifugation. The verification procedure employed the first step of the HRV analysis described in the latter part of the above-mentioned section “Study Protocol.” More specifically, the identification of the R peaks resulted in the normalized heart rate which computed as the average number of R peaks per minute. Then the ratio of the heart rate divided by (220-age) was used to estimate whether the exercise training could be regarded as light intensity.

## Results

### Motion Sickness and Comfort Results

No soreness other than normal exercise fatigue was reported. None of the participants reported an overall motion sickness rating higher than 1 in a 0–10 scale and only one reported slight symptom (scale 1) due to momentarily moving his head to the opposite side. The spin-down process caused transient lightheadedness, due to the sensation of moving counter clock-wise during slowing down to stop spinning (Duda et al., 2012), but lasted only several seconds After that, the participants were allowed to remove their eye cover for the rest of the waiting time and during the wash-out periods.

### Gender Analysis

A 2 (gender: male vs female) × 2 (condition: standing vs AG training) between subjects’ ANOVA was conducted to study cardiovascular variables differences (CO, CI, CP, MAP, SBP, DBP, HR, Class, Fmax, VHF, VLF, LF, HF, and LF/HF) between male and female participants. There was a statistically significant main effect of gender in CO and CP biomarkers (see Table 2). A statistically significant gender × condition interaction was observed only in the HF biomarker (see Table 3). The AG training was set as the mean biomarker value for the seven (0.5, 0.7, 1.0, 1.2, 1.5, 1.7, 2.0g) AG training loads.

**TABLE 3:** Description of the biomarkers that showed a statistically significant gender by experimental condition interaction when comparing the AG training levels with the standing condition.

Biomarker	Interaction significance	*Post hoc* comparison	Descriptive statistics
HF	*F*(1,10) = 7.314, *p* = 0.022	Female (standing – AG training) / *p* = 0.004	Standing (0.49 ± 0.10); AG training (0.37 ± 0.08)

In order to further investigate this interaction, we conducted Bonferroni *post hoc* comparisons. It indicated a statistically significant effect only for females (*p* = 0.004). More specifically, females showed decreased HF values during the AG training in comparison with the standing condition (see Table 3 for more details).

We further investigated which specific AG task was responsible for this decrease. So, we performed non-parametric analysis for the female participants comparing the standing condition with each AG load separately. The Friedman test showed a marginally significant effect of the AG level on HF on female participants (χ^2^(7) = 12.410, *p* = 0.088). Wilcoxon tests were used to follow-up these findings.

Since, we performed multiple comparisons among the standing condition and the seven AG loads, a Bonferroni correction was applied and so all effects are reported at a *p* = 0.007 (0.05/7) level of significance. It appeared that there were only marginal statistical differences, since none of them survived the multiple comparisons’ correction: (a) from standing to 0.5g (*Z* = −2.490, *p* = 0.013), (b) from standing to 1.0g (*Z* = −2.223, *p* = 0.026), (c) from standing to 1.7g (*Z* = −2.401, *p* = 0.016), and (d) from standing to 2.0g (*Z* = −2.223, *p* = 0.026).

### Cardiovascular and Blood Gas Analysis

Cardiovascular and blood gas analysis variables were examined in graded g loads (0.5, 0.7, 1.0, 1.2, 1.5, 1.7 and 2.0g) and mean values and standard deviations of all measured values are depicted in Table 4.

**TABLE 4:** Mean (*M*) ± Standard Deviations (SD) of 15 biosignal features for the five AG levels, where the high intensity level due to its significance compared to standing, it was presented separately as 1.7 and 2.0g.

Biosignal features			AG levels (g)				
	Standing 5_min	Ultra-low intensity (0.5g)	Low intensity (0.7 and 1g)	Medium intensity (1.2 and 1.5g)	High intensity (1.7 and 2g)	1.7g	2g
*_HR_*	83.42 ± 11.82	75.92 ± 12.16	75.27 ± 10.48	75.58 ± 9.97	78.17 ± 9.42	78.46 ± 11.26	78 ± 10.21
*_CO_*	7.21 ± 1.88	6.07 ± 1.26	6.02 ± 1.27	6.20 ± 1.29	6.33 ± 1.75	6.18 ± 1.83	6.47 ± 1.79
*_MAP_*	88.42 ± 10	82.58 ± 8.44	83.60 ± 8.55	86.04 ± 7.87	87.30 ± 9.35	87.60 ± 9.85	86.79 ± 10.07
*_CI_*	3.90 ± 1	4.19 ± 4.28	3.26 ± .69	3.83 ± 2.23	3.43 ± .93	3.35 ± 0.98	3.50 ± 0.94
*_CP_*	1.44 ± 0.49	1.40 ± 1.27	1.14 ± 0.34	1.40 ± 0.74	1.24 ± 0.44	1.22 ± 0.45	1.28 ± 0.47
*_SBP_*	125.54 ± 11.91	121.04 ± 16.65	121.63 ± 8.67	124.79 ± 9.84	126.29 ± 11.70	127.13 ± 10.58	125.08 ± 14.60
*_DBP_*	75.75 ± 8.68	72.08 ± 10.30	75.04 ± 6.79	77.29 ± 7.85	78.44 ± 9.60	78.33 ± 9.35	78.17 ± 11.35
*_SV_*	84.13 ± 14.29	82.63 ± 14.32	82.26 ± 15.74	85.57 ± 16.17	85.62 ± 16.97	83.97 ± 16.57	87.19 ± 18.78
*_TPR_*	12.92 ± 3.19	13.43 ± 3.34	14.21 ± 1.81	13.97 ± 2.19	14.49 ± 2.80	14.96 ± 3.34	14.03 ± 2.65
*_pH_*	7.37 ± 0.02	7.36 ± 0.02	7.36 ± 0.01	7.36 ± 0.02	7.36 ± 0.03	7.35 ± 0.04	7.36 ± 0.03
*_pO_2_*	92.46 ± 5.48	91.75 ± 4.76	91.96 ± 3.77	92.35 ± 2.72	91.50 ± 4.13	91.58 ± 5.22	91.38 ± 4.63
pCO_2_	42.63 ± 3.08	42.58 ± 2.76	42.50 ± 2.68	41.17 ± 2.57	41.67 ± 3.11	42.04 ± 3.25	41.25 ± 3.08
*_O_2 ^–_*	16.53 ± 1.46	15.80 ± 2.48	16 ± 1.72	16.42 ± 1.36	16.07 ± 1.60	16.05 ± 1.87	16.09 ± 1.87
*_CO_2 ^–_*	28.25 ± 4.60	27.92 ± 3.49	27.75 ± 4.12	26.02 ± 2.80	26.29 ± 3.35	26.96 ± 3.53	25.63 ± 3.84
*_O_2_*sat*%_*	95.33 ± 2.14	95.59 ± 2	95.42 ± 1.27	95.85 ± 1.07	95.46 ± 2.46	95.54 ± 1.79	95.38 ± 3.45

**FIGURE 2:**
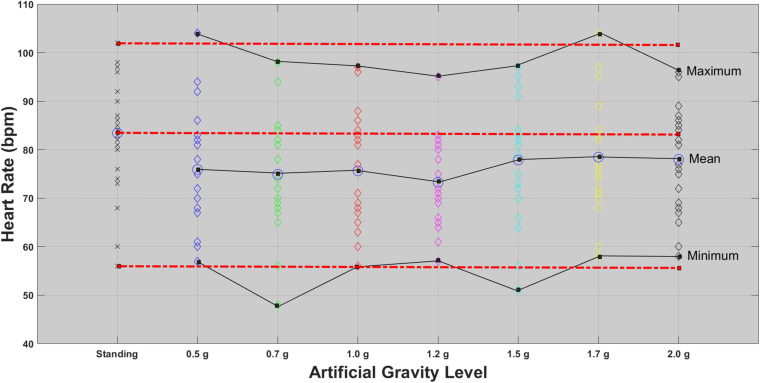
HR for each of the 24 participants in the standing position (red dotted line) and at each artificial gravity (AG) load (denoted with a diamond of a different color: blue, 0.5g; green, 0.7g; red, 1.0g; magenta, 1.2g; cyan, 1.5g; yellow, 1.7g; black, 2.0g).

A one-way repeated-measures ANOVA was conducted with five intensity levels (standing 5 min, ultra-low intensity, low intensity, medium intensity, and high intensity) as the within-subjects factor on HR, pO_2_, pCO_2_, and ${\text{O}}_{2}^{--}$ which served as the dependent variables. Assumptions of normality using the Kolmogorov–Smirnov test and homogeneity of variance for homoscedasticity using a Levene’s test were met (see Figure 2).

### Heart Rate Variability

Investigating the temporal variation between each heartbeat, HRV revealed a statistically significant main effect of AG levels for Class $\left[F_{\left(7,147\right)}=3.500,p<0.01,\eta_{p}^{2}=0.143\right]$, Fmax $\left[F_{\left(7,147\right)}=4.467,p<0.01,\eta_{p}^{2}=0.175\right]$ and VHF $\left[F_{\left(7,147\right)}=4.492,p<0.01,\eta_{p}^{2}=0.176\right]$ (Table 5). Performing Bonferroni *post hoc* procedure, pairwise comparisons indicated a trend to significant mean difference for Class between:

(i) Standing 5 min and 1.5g (*mean difference* = −0.378, *p* = 0.035, *CI* (95%) −0.740, −0.015) and
(ii) Fmax between standing 5 min and 0.5g (*mean difference* = −0.118, *p* = 0.026, *CI* (95%) −0.228, −0.008),
(iii) Standing 5 min and 0.7g

$$\begin{array}{c} (meandifference=-0.104,p=0.005,CI\left(95\%\right)\\ -0.185,-0.023)and \end{array}$$

(iv) Standing 5 min and 1.2g

$$\begin{array}{c} (meandifference=-0.095,p=0.024,CI\left(95\%\right)\\ -0.183,-0.008)and \end{array}$$

**FIGURE 3:**
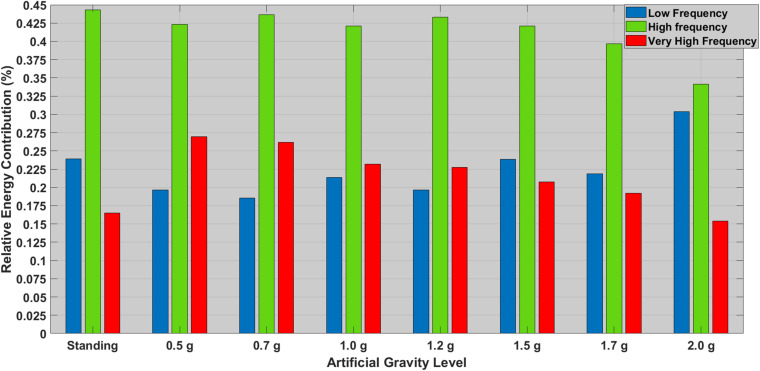
Relative contribution (%) of the most significant HRV features between standing and all AG loads. The low frequency (LF) ranges is denoted with a blue bar (0.04–0.15 Hz), the high frequency (HF) is denoted with a green bar (0.15–0.40 Hz), and the very high frequency is denoted with a green bar (>0.40 Hz).

(v) Standing 5 min and 1.5g

$$\begin{array}{c} (meandifference=-0.083,p=0.011,CI\left(95\%\right)\\ -0.154,-0.012) \end{array}$$

(Table 5 *and* Figure 3).

(vi) No significant difference was revealed between standing and 1.7 and 2.0g for class and Fmax.

**TABLE 5:** Visualization of the spectral heart rate variability (HRV) features that yielded statistically significant results due to the artificial gravity (AG) training.

Spectral property	F-value	*p*-Value	Effect size	
Class	3.500	0.002	0.143	
Fmax	4.467	0.000	0.175	
VHF	4.492	0.000	0.176	
	**Pairwise comparisons**	**Mean difference**	***p*-Value**	**CI (95%)**
Class	Standing 5 min vs 1.5g	−0.378	0.035	(−0.740, −0.015)
Fmax	Standing 5 min vs 0.5g	−0.118	0.026	(−0.228, −0.008)
Fmax	Standing 5 min vs 0.7g	−0.104	0.005	(−0.185, −0.023)
Fmax	Standing 5 min vs 1.2g	−0.095	0.024	(−0.183, −0.008)
Fmax	Standing 5 min vs 1.5g	−0.083	0.011	(−0.154, −0.012)
VHF	Standing 5 min vs 0.7g	−0.108	0.019	(−0.206, −0.011)
VHF	0.7 vs 2g	0.105	0.012	(0.015, 0.195)

*These features were the maximum frequency (Fmax) of the Lomb–Scargle periodogram, its corresponding frequency range (Class) and the very high frequency (VHF) range.*

A significant mean difference of AG levels for VHF was revealed between:

(i) Standing 5 min and 0.7g

$$\begin{array}{c} (meandifference=-0.108,p=0.019,CI\left(95\%\right)\\ -0.206,-0.011)and \end{array}$$

(ii) A total of 0.7 and 2.0g

$$\begin{array}{c} (meandifference=0.105,p=0.012,\\ CI\left(95\%\right)0.015,0.195), \end{array}$$

suggesting the activation of the parasympathetic system at 0.7g initially and then at 2.0g, indicating the effectiveness of the centrifuge as training device.

For the pairwise comparisons, all the mean differences were significant at the 0.05 level, adjusted for multiple comparisons: Bonferroni. These results and their statistical significance are described in Table 6.

**TABLE 6:** Description of the heart-rate related exercise intensity ratio that showed a statistically significant a main effect of condition when comparing the AG training levels between them.

	*F*-value	*p*-Value	Effect size
Main effect of exercise intensity ratio	9.331	0.000	0.318

**Pairwise comparisons**	**Descriptive statistics**	**Mean difference**	**p-value**

0.5G–1.5G	0.5G (0.43 ± 0.05); 1.5G (0.48 ± 0.05)	−0.055	0.045*
0.5G–1.7G	0.5G (0.43 ± 0.05); 1.7G (0.50 ± 0.05)	−0.076	0.001***
0.5G–2G	0.5G (0.43 ± 0.05); 2G (0.49 ± 0.06)	−0.069	0.011**
0.7G–1.7G	0.7G (0.44 ± 0.06); 1.7G (0.50 ± 0.05)	−0.066	0.000***
1G–1.7G	1G (0.45 ± 0.05); 1.7G (0.50 ± 0.05)	−0.049	0.048*

*These results will not survive the multiple comparisons’ correction. **These results will marginally survive the multiple comparisons’ correction. ***These results will survive the multiple comparisons’ correction.

The pairwise comparisons of the maximum HRV frequency (Fmax) demonstrated a statistically significant increase between Standing when compared to 0.5g (*p* = 0.026), 0.7g (*p* = 0.005), 1.2g (*p* = 0.024), and 1.5g (*p* = 0.011), but no statistically significant difference between 1.7 and 2.0g, indicating that those loads were closer to standing.

Concerning VLF, LF, HF, and LF/HF, as assumptions of one-way repeated-measures ANOVA were violated, a non-parametric Friedman’s ANOVA was used for comparison of the eight AG levels on the abovementioned spectral properties. Furthermore, non-parametric *post hoc* procedures conducted using Wilcoxon signed-rank tests, but correcting for the number of comparisons by accepting something as significant only if its significance is less than a/number of comparisons (the Bonferroni correction). In this analysis, due to 8 AG levels (standing plus 7 AG levels), we have 28 comparisons, therefore, rather than use 0.05 as our critical level of significance, we had use 0.05/28 = 0.002. The Friedman test showed significant effect of AG levels on LF (χ^2^(7) = 15.833, *p* = 0.027) and LF/HF (χ^2^(7) = 17.045, *p* = 0.017) and marginal main effect for VLF (χ^2^(7) = 13.833, *p* = 0.054), and HF (χ^2^(7) = 5.909, *p* = 0.550). These results and their statistical significance are described in the Supplementary Material.

As shown in Figure 3, both standing condition and 2.0g AG induce greater sympathetic (LF) and lesser parasympathetic (HF) response. The VHF ratio is also lower for these two conditions. Wilcoxon tests were used to follow-up these findings. A Bonferroni correction was applied and so all effects are reported at *a* = 0.002 level of significance. It appeared that: (i) LF significantly increased from 0.7 to 2.0g (*Z* = −3.376, *p* = 0.001) and (ii) LF/HF significantly increased from 0.7 to 2.0g (*Z* = −3.406, *p* = 0.001), indicating the effectiveness of the centrifuge as training device.

**FIGURE 4:**
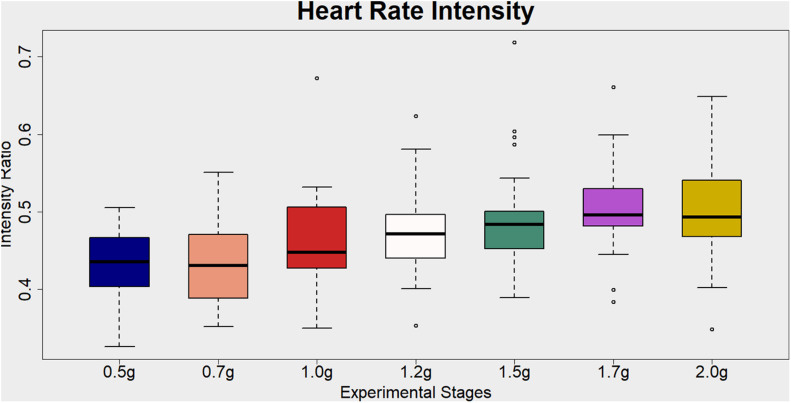
Visualization of the Heart Rate Intensity ratio (%) for each artificial gravity (AG) condition. This is a cardiovascular index of the exercise intensity level. It is estimated as the heart rate (beats per minute) during each training phase divided by the maximum heart rate (MHR) intensity. The MHR for each participant is defined as 220-participant’s age. Ratio values ranging from 40 to 59% are indicative of light exercise.

A repeated measure of ANOVA with a Greenhouse–Geisser correction and condition as within subjects’ factor (0.5, 0.7, 1, 1.2, 1.5, 1.7, and 2g) was conducted to study heart-rate related exercise intensity ratio differences among the aforementioned seven AG levels. There was a statistically significant main effect of condition [*F*(3.913,120) = 9.331, *p* < 0.001] (see Figure 4 and Table 6 for more details and *post hoc* comparisons).

### Blood Gas Analysis

No significant main effect was revealed comparing the different g loads for pH, ${\text{CO}}_{2}^{--}$, pCO_2_, ${\text{O}}_{2}^{--}$, and O_2_sat%. Comparing standing with 1.7 and 2.0g with the Friedman test showed significant effect of AG load on pCO_2_ (χ^2^(2) = 7.342, *p* = 0.025) and a marginal trend toward a significant main effect on (${\text{CO}}_{2}^{--}$)(χ^2^(2) = 5.948, *p* = 0.051). Wilcoxon tests were used to follow-up these findings. A Bonferroni correction was applied and so all effects are reported at a 0.016 (0.05/3) level of significance. It appeared that (${\text{CO}}_{2}^{--}$) significantly decreased from 1.7 to 2.0g (*Z* = −2.433, *p* = 0.015). In addition, if a Bonferroni correction is not applied so the level of significance will be at 0.05, then, we observe that pCO_2_ significantly decreased: (i) from standing to 2.0g (*Z* = −2.034, *p* = 0.042) and ii) from 1.7 to 2.0g (*Z* = −2.171, *p* = 0.030). So, by increasing the AG level of 1.7–2.0g we propose that the respiration rate increased and consequently the mean value of pCO_2_ dropped. Note a substantial decrease of pCO_2_ at 1.2g where the baroreceptors were triggered.

### Cardiac Output, Cardiac Index and Cardiac Power Output

**FIGURE 5:**
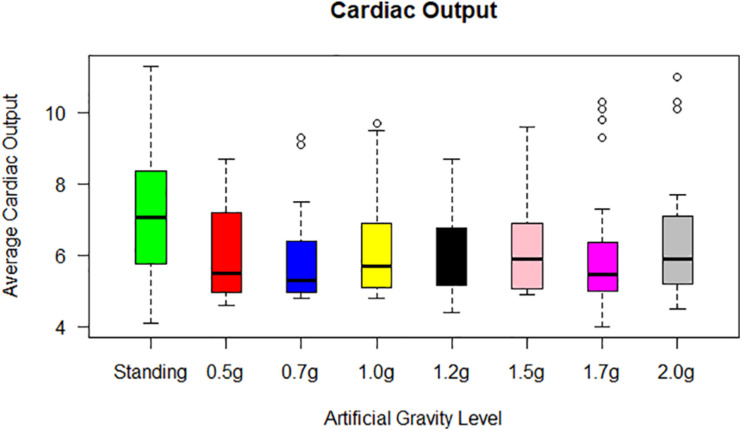
Cardiac output (CO) for each of the 24 participants in the standing position (green) and at each artificial gravity (AG) load (denoted with a boxplot of a different color: red, 0.5g; blue, 0.7g; yellow, 1.0g; black, 1.2g; pink, 1.5g; magenta, 1.7g; gray, 2.0g).

The Friedman test showed significant effect of the AG load of CO (χ^2^(4) = 19.713, *p* = 0.001). Wilcoxon tests were used to follow-up these findings. It appeared that:

(i) CO significantly decreased: (a) from standing to ultra-low intensity (*Z* = −3.651, *p* = 0.000), (b) from standing to low intensity (*Z* = −3.346, *p* = 0.001), (c) from standing to medium intensity (*Z* = −3.258, *p* = 0.001) and (d) from standing to high intensity (*Z* = −2.844, *p* = 0.004) (Figure 5),
(ii) CI significantly increased from standing to ultra-low intensity (*Z* = −3.011, *p* = 0.003) and significantly decreased: (a) from standing to low intensity (*Z* = −3.285, *p* = 0.001) and from (b) standing to high intensity (*Z* = −2.857, *p* = 0.004),
(iii) Cardiac power significantly decreased: (a) from standing to ultra-low intensity (*Z* = −3.143, *p* = 0.002), (b) from standing to low intensity (*Z* = −3.157, *p* = 0.002), and (c) DBP significantly increased from low intensity to high intensity (*Z* = −2.937, *p* = 0.003).

The Friedman test showed a significant effect of AG level on CO (χ^2^(2) = 15.432, *p* = 0.000), CI (χ^2^(2) = 15.432, *p* = 0.000), CP (χ^2^(2) = 8.374, *p* = 0.015). Wilcoxon tests were used to follow-up these findings. A Bonferroni correction was applied and so all effects are reported at a 0.016 (0.05/3) level of significance. It appeared that:

(i) CO significantly decreased from standing to 1.7g (*Z* = −3.318, *p* = 0.001),
(ii) CI significantly decreased from standing to 1.7g (*Z* = −3.429, *p* = 0.001),
(iii) CP significantly decreased from standing to 1.7g (*Z* = −2.571, *p* = 0.010).

None or only minor changes were seen in the mean SV level during the exercise periods. Upon the end of exercise, a striking but transient increase in CO occurred, resulting from an increase in SV concomitant with a maintained HR (Figure 5). Converting CO to CI (CO/BSA), similar findings were revealed with an increase towards the higher g loads.

### Mean Arterial Pressure

**FIGURE 6:**
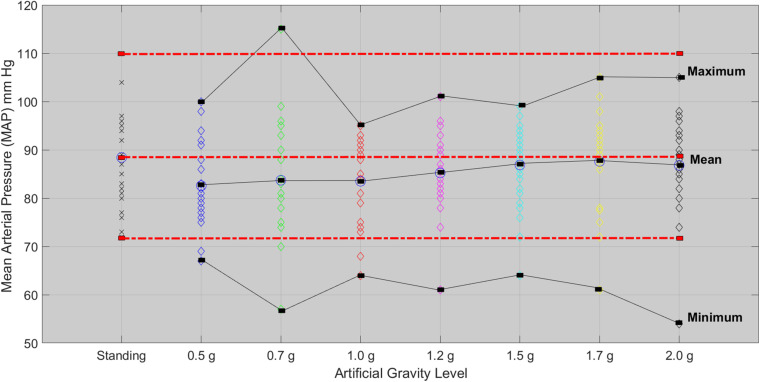
Mean arterial pressure for each of the 24 participants in the standing position (red dotted line) and at each artificial gravity (AG) load (denoted with a diamond of a different color: blue, 0.5g; green, 0.7g; red, 1.0g; magenta, 1.2g; cyan, 1.5g; yellow, 1.7g; black, 2.0g).

The Friedman test showed significant effect of AG level on MAP (χ^2^(4) = 16.201, *p* = 0.003). A second Friedman’s ANOVA was used to compare standing with 1.7g and 2.0g and there was no statistically significant difference. Both 1.7 and 2.0g loads of Art G result in a non-significant change in MAP with respect to standing (Figure 6).

### Systolic Blood Pressure and Diastolic Blood Pressure

**FIGURE 7:**
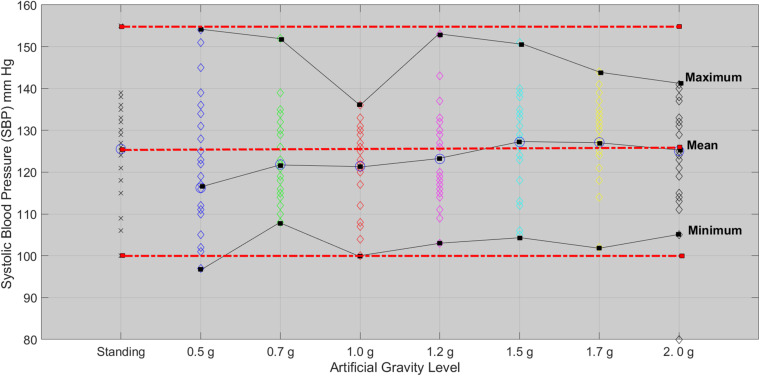
Systolic blood pressure for each of the 24 participants in the standing position (red dotted line) and at each artificial gravity (AG) load (denoted with a diamond of a different color: blue, 0.5g; green, 0.7g; red, 1.0g; magenta, 1.2g; cyan, 1.5g; yellow, 1.7g; black, 2.0g).

The Friedman test showed significant effect of the five AG level on SBP (χ^2^(4) = 9.595, *p* = 0.048). Wilcoxon tests were used to follow-up these findings. A Bonferroni correction was applied and so all effects are reported at a 0.005 level of significance. SBP marginally significant decrease from high intensity to low intensity (*Z* = −2.647, *p* = 0.008). A second Friedman’s ANOVA test comparing the standing with 1.7 and 2.0g did not show any statistical significance for SBP and DBP, which means that 1.7 and 2.0g results in a non-significant change in BP with respect to standing. SBP followed the MAP and leveled off after 1.7g (Figure 7).

### Correlation Results

**FIGURE 8:**
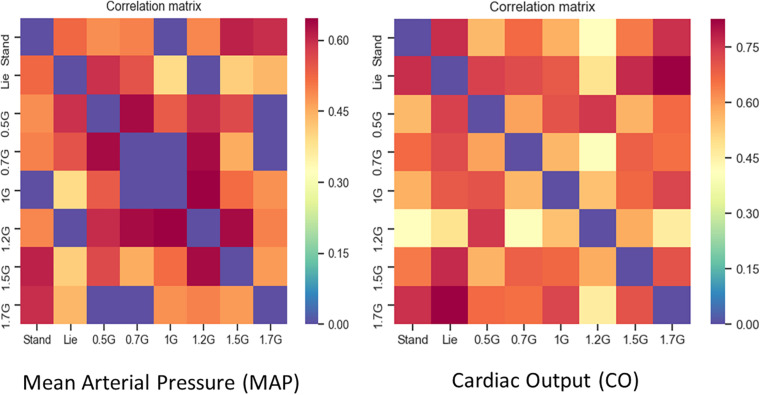
Correlation Matrices for MAP **(left picture)** and CO **(right picture)**. ρ values with no statistical significance (*p* > 0.05) were set to zero (blue). Furthermore, the main diagonal of the matrix was set to zero (blue) as well. Dark red in the correlation matrix represents the higher ρs (0.7 for MAP and 0.8 for CO), blue represents the zeros, while the rest of the colors all values between.

From the correlation matrix (Figure 8) of the hemodynamic parameters of CO and MAP standing significantly correlated with 1.7 and 2.0g. This enhances the significance of those parameters to construct dose response curves and also determine the optimal g load for training similar to standing condition along the Gz axis.

### Dose Response Curves

**FIGURE 9:**
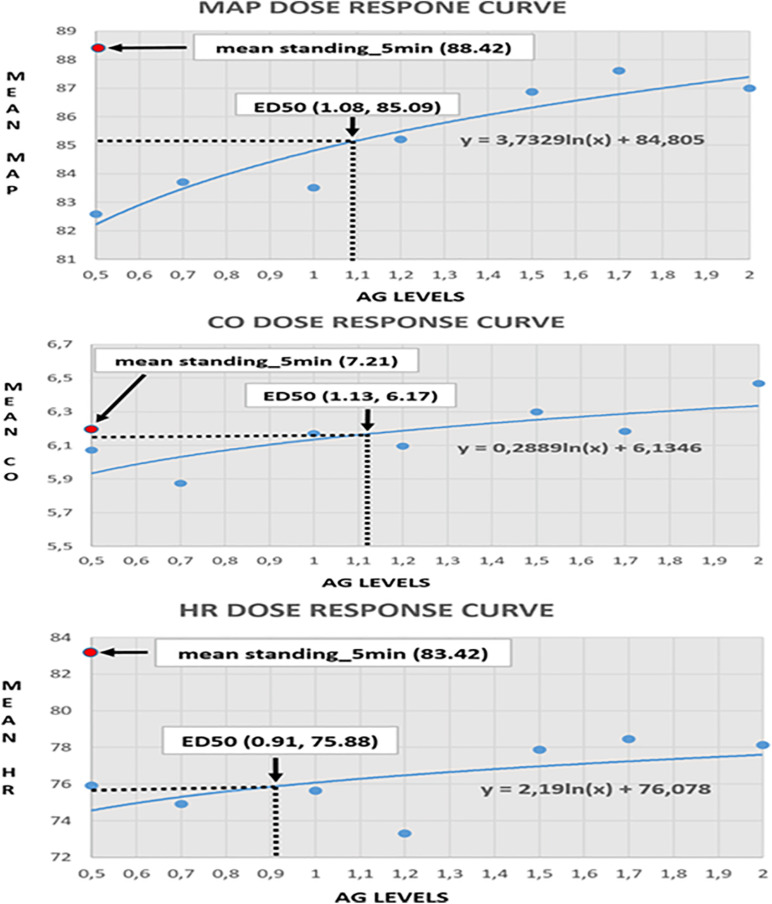
Visualization of the dose-response curves for mean aretrial pressure (MAP), cardiac output (CO), and heart rate (HR) in seven AG loads (0.5, 0.7, 1, 1.2, 1.5, 1.7, and 2g). The MAP-ED50 is about 1.08g, corresponding to a mean MAP of 85.09. The CO-ED50 is about 1.13g, corresponding to a mean CO of 6.17. The HR_ED50 is about 0.91g, corresponding to a mean HR of 75.88.

Clinical monitoring of the significant cardiovascular variables during centrifugation at graded Gz from 0.5 to 2.0g enabled the construction of dose response curves. These were constructed from measurements of MAP, CO and HR (Figure 9) in all 24 young healthy participants (12 male and 12 females) studied at seven different loads of AG 0.5, 0.7, 1.0, 1.2, 1.5, 1.7, and 2.0g. Furthermore, the median effective dose at which 50% of subjects exhibit the specified biomarker effect is calculated (ED50), which constitutes a useful guide for determining the appropriate G load for training in conditions that are similar to those of standing along the Gz axis (Figure 9). In addition, ED50 enables the selection of the most useful and safer G load, avoiding undermining morbidity, particularly in those induced by inactivity in bedrest, by various mobility disorders or to mitigate detrimental effects of microgravity in astronauts. Comparing CO, MAP, and HR under seven AG loads (0.5, 0.7, 1.0, 1.2, 1.5, 1.7, and 2.0g), we found increased levels of HR, CO, MAP, and in high loads of AG. The ED50 was about 1.08g for MAP, 1.13g for CO, and 0.91g for HR, respectively.

## Discussion

The present study provided insight into determining the effective Gz suitable for training or rehabilitation, focusing mainly on the cardiovascular system. In order to come to this conclusion, data from 24 healthy volunteers were collected in seven different loads of AG (0.5, 0.7, 1.0, 1.2, 1.5, 1.7, and 2.0g). The data included SBP, DBP, MAP, CO, SV, HR, pO_2_, pCO_2_, O_2_^–^, CO_2_^–^, and O_2_sat%. The main findings involved the detection of g load equivalent to standing for hemodynamic variables and the construction of dose response curves for the most significant ones. The study was designed to begin to systematically build the framework for the study of gravity physiology, its threshold requirements, as well as its potential uses in training or rehabilitation therapy.

Standing on Earth is crucial for the maintenance of all physiological systems. According to the present study, standing equivalent benefits, assumed to be 1Gz, in microgravity analog environments or in the case of immobilized patients, can be achieved by the centripetal acceleration in levels higher than 1.5g in order to maintain cardiovascular function. For some cardiovascular variables, even centrifugation at 2g was not enough to reach comparable standing values, while others needed at least 1.7g.

In this study the spectral features of heart-rate variability analysis were investigated through the LSP, which demonstrated significant effects for a variety of features such as Class, Fmax, VHF, LF, and LF/HF. Based on the study of Migeotte et al. (2009), who underlined the need for further investigation on the changes in graded g load and the gravity gradient in order to find the appropriate g load for SAHC countermeasure, we aimed to examine whether the SAHC training could induce cardiac responses analogous to the standing position. HRV, which has been established during gravity transitions (Sasaki et al., 1999; Seps et al., 2002) was one of the tools used among the cardiovascular indices, to investigate the control and modulation of cardiovascular function through the autonomic nervous system during graded Gz (Malik, 1998). Rowell reported that the changes of HRV in the standing condition are mainly due to the sub-diaphragmatic venous pooling (Rowell, 1993), resulting in a decrease of venous return to the right atrium and the CO in standing and supine position during centrifugation. The higher LF component in the standing position suggest not only higher vagal tone, but also an increment in sympathetic tone, leading to sympathovagal balance decrease due to these changes of vagal activity. The pairwise comparison analysis for most of the features, identified significant differences between the standing position when compared to low and medium intensity level centrifugation (0.5, 0.7, 1.2, and 1.5g). Non-parametric analysis for sympathetic (LF and LF/HF) indices showed significant differences among 2.0 and 0.7g. These results indicate that AG training altered the HRV spectral properties. More specifically, the main effect of Fmax demonstrates that AG training induced a statistically significant shift of the frequency content among the standing and the low and medium AG intensity (0.5/0.7/1.2/1.5g). However, no statistically significant difference was found between standing when compared to the 1.7 and 2.0g. Figure 3 shows that both the standing condition and the 2.0g AG induces greater sympathetic (LF) and lower parasympathetic (HF) contribution. This implies that these specific AG levels are similar to the standing position. Moreover, analysis of the autonomic markers of the sympathetic activity (LF and the ratio of LF/HF) showed that there was a statistically significant increase at 2.0g (*p* = 0.011), when compared to the 0.7g. This finding implies that the 2.0g induces shorter and more uniform (less varied) R–R intervals due to increased sympathetic activity and simultaneous parasympathetic withdrawal. Also, it demonstrates a similarity with what happens during standing, where BP is regulated by low afferent discharge of the baroreceptor in the aortic arch and carotid sinus, leading to increased HR due to vagal withdrawal and sympathetic nerve activation (Smith et al., 1994; Heesch, 1999; Verma et al., 2018). AG training at 2.0g causes increased physiological stress and elevated fatigue level in comparison to the 0.7g (Makivić et al., 2013). Similarly, there were statistically significant changes among the 0.7g condition when compared to the standing (*p* = 0.019). Migeotte et al. (2009) reported that the change of HRV in hypergravity are due to venous pooling reduced filling of the right atrium and autonomic adaptation. The major point to be made is that VLF, as the sympathetic nervous system was activated, increased with the gradual increase of g load and was followed by a rapid increase of MAP by constricting arterioles, followed by increased heart rate and SV (Figure 2 and Table 4). Additionally, the higher muscle metabolism causes vasodilatation and therefore peripheral resistance decreases.

In our healthy population cohort, there was a statistically significant increase of VHF relative energy contribution, which denotes activation of the parasympathetic nervous system and the baroreceptor reflex responses to changes in arterial pressure, which resulted from the transition from 0.5 to 0.7g (*p* = 0.019) CI (95%) and then from 1.7 to 2g (*p* = 0.012) CI (95%) with a simultaneous decrease in heart rate and CO. These cardiovascular findings are in line with previous studies (Wehrwein and Joyner, 2013) indicating a position change (supine to upright). More specifically, it involves both increased atrial pressure and afferent firing regarding the carotid sinus nerve. These alterations attenuated sympathetic and increased parasympathetic activity. The opposite pattern occurs in cases of decreased arterial pressure.

The HRV findings validate our hypotheses that (a) both standing and 2.0g AG training share common HRV spectral patterns and (b) induce a much greater sympathetic tone than the ultra-low (0.7g) AG intensity. These results extend preliminary knowledge obtained by recent studies which indicated that 2.0g centrifugation shares more similarities with the Standing condition than the 1.0g level (Verma et al., 2018). Moreover, standing seems to induce a shift in the maximum HRV frequency in comparison to all AG levels other than those greater than 1.0g. The higher LF component within the standing position suggest not only higher vagal tone, but also an increment in sympathetic tone. Due to these changes in vagus nerve activity, sympathetic balance will decrease.

Statistical analysis revealed a significant main effect of AG load on the cardiovascular variables of HR, MAP, CO, CI, CP, and SBP. A significant effect of AG intensity level for heart rate was observed compared to standing with all g loads except high intensity load of 1.7 and 2.0g. Furthermore, pairwise comparisons among standing and 1.7 and 2g showed no statistically significant difference between 1.7g and standing, while it was marginally significantly different at 2g. SBP (Figure 7) and DBP dropped in 0.5g and increased significantly at 0.7g and higher g levels. The application of AG introduces a new stress condition that the cardiovascular system needs to overcome in order to assure the appropriate amount of blood flow to all parts of the body. This additional stress is reflected on the BP drop that occurs when the participant is first exposed to AG. Our findings (Table 5) indicate that the increased centrifugation intensity induced greater sympathetic activity through subsequent increases in heart rate and peripheral resistance. The increased g-load raises the need for elevated CO and vasoconstriction in order to facilitate both venous and BP return to normal level at the baroreceptors. This is a physiological compensatory mechanism against high leg pressure and subsequent increased orthostatic challenge. An alternative of this mechanism may be the inclusion of the cycling exercise which acts as a mechanical pump assisting venous return by reducing the blood pooling in the legs and the orthostatic challenge. This was firstly demonstrated a decade ago by pioneering studies (Clément and Bukley, 2007; Kaderka et al., 2010). We adopted a similar design in our study in order to enhance the beneficial role of the proposed intervention. Consequently, CO also increases to meet the new metabolic demands imposed by exercise. The higher AG combined to workload, result in higher CO, which significantly changed at all g intensity levels. Comparing standing with 1.7 and 2g there was a statistically significant difference with 1.7g but not with 2.0g, suggesting that 2.0g load was more comparable to standing (Figure 5). The mean CO value in the standing position (7.21) is higher than any mean value obtained during centrifugation. The lowest CO mean value during centrifugation was obtained at 0.7g (6.07) and the highest at 2.0g (6.46) (Figure 5). There is no significant difference between minimum CO values at any art G load. At 1.7 and 2.0g, maximum CO was restored to that of standing (Figure 5). The same applies to CI and CP.

When comparing MAP, SBP, and DBP between standing, 1.7 and 2g, there were no statistically significant differences, suggesting that MAP may indicate similar effects to standing in both 1.7 and 2.0g loads. From the correlation matrix for CO, the data reveal a strong correlation of standing with 1.7g (0.77) and 0.5g with lying down (0.92). Additionally, covariance was calculated at a positive value. Therefore, CO is a good indirect marker, indicative of a beneficial effect of g load on the human body (Figure 5).

We also observed a main effect of gender on CO and CP and a gender by condition interaction regarding the parasympathetic index of HF of the HRV activity. Although, we should be cautious due to the small sample size and the case of marginally statistically significant differences when correcting the significance level at *p* = 0.007 to avoid Type-1 errors, these gender differences may be due to the parasympathetic withdrawal response during the AG training when compared to the standing condition. Our hypothesis, which should be further tested by subsequent studies is that due to vagal parasympathetic withdrawal, HR was increased abruptly at the onset of exercise and progressively continued to increase during exercise because of a combination of sympathetic neural and humoral drive, and quickly returned to baseline after cessation of exercise because of withdrawal of neural sympathetic discharge. Women responded differently with increased parasympathetic withdrawal as reported in previous studies (Evans et al., 2001). The difference was attributed to the autonomic function, as well as other factors may contribute, such as myocardial structure, lower SV and hormonal status. The gender difference in orthostatic stress has been demonstrated in other studies as well (Hart and Charkoudian, 2014), with women responding with increased HR while men responded with an increase in vascular resistance (Shoemaker et al., 2001), or increase in HR, DBP, and SVRI (Masatli et al., 2018). Additionally, women presented greater baroreflex sensitivity than men (Drudi and Grenon, 2014). From research in Space, it is known that women are more prone to OI when returning from Space (Wenner et al., 2013) and a need for more effective countermeasure for cardiovascular deconditioning was always a priority for both genders of the human research roadmap (Vernikos et al., 2016) with particular interest in cardiovascular responses of women since this has not been thoroughly documented during intermittent AG exposure so far. Although the number of participants in this study was limited, one of the findings of this study was that the +Gz gradient required in order to elicit significant increases in cardiovascular responses was different and need further investigation to control for significant baseline anthropometric gender differences.

We should also highlight here the wide-range, dose response curves constructed from healthy individuals. These add to the knowledge obtained so far from previous studies where selective g levels of 1.0 and 2.0g were studied only without exercise (Goswami et al., 2015; Masatli et al., 2018; Verma et al., 2018), or dose response curves were generated by regression models since selective Gz of 1 G, and 1.4 G at the feet were used combined with different intensity exercise levels (Diaz-Artiles et al., 2018). To fill in this gap and better understand the relationship between gravitational dose and physiological response (Clément, 2017), dose response curves were constructed based on all intermediate stages tested from 0.5 to 2g in both men and women combined with mild intensity exercise well controlled by HR and HRV. The novelty of this work was the range of gravity levels (0.5, 0.7, 1.0, 1.2, 1.5, 1.7, and 2.0g) studied. The use of standing as the ground reference of Gz on Earth and the comprehensive database of cardiovascular variables (CO, CI, CP, MAP, SBP, DBP, HR, Class, Fmax, VHF, VLF, LF, HF, and LF/HF) analyzed, led to the construction of the dose-response curves. Although the analysis focused on healthy men and women, it is the authors’ goal and future plan to extend the application of the SAHC and continue to build and apply the data base in other age groups of healthy participants, as well as in the rehabilitation of patients suffering from various diseases and conditions, that restrict mobility or confine them to bed.

In summary, the present study was designed initially to introduce and validate a new SAHC device in Greece, to construct a reference framework required to base choices and decisions in research, in identifying optimal G load requirements for patients during AG therapy, for physical therapy and rehabilitation or for use of optimal AG as a spaceflight countermeasures. Mobility is the single crucial element underlying healthy living and healthy aging, as mobility on Earth is gravity-dependent.

Our encouraging findings should be considered in the context of several limitations. First of all, the use of Clearsight monitor to record non-invasive hemodynamics, which offer an estimate measure of CO based on internal models. These devices have a Height Reference that is supposed to be connected at heart level to counteract hydrostatic change (for example, when the participants move their hand where the cuffs are connected). We have decided to not use this correction, as other researchers (Goswami et al., 2015) did before, but instead, we asked all participants to keep their hand at heart level and not move for the entire centrifugation protocol. Another limitation is the Exercise protocol where although we measured light exercise according to % MHR and Carvonen formula and verified by continuous ECG measurement and HRV, we did not measure exactly the force exerted on the ergometer. The number of participants was also limited, especially for female participants, borderline to achieve significance, although sample size was similar to what was used in previous studies that examined mixed sex population during central hypovolemia testing (Evans et al., 2015, 2018; Goswami et al., 2019). Finally, we should also note that the proposed protocol could not reproduce normal standing condition since most people spend about 16 h/day in upright posture.

## Conclusion

We introduced a new Short Arm Human Centrifuge in Greece with the first experiment to be conducted, demonstrating good operations and feasibility of scientific experiments, where subjects tolerated well the protocol without any undesired effects. Our main findings approached an answer to the key question of the analogous to standing level of g. We also identified the cardiovascular markers and their dose response curves, providing the answer to the appropriate configuration for individual training and rehabilitation due to pathological conditions which might be proved to be the novel therapeutic physiological approach for people with a broad spectrum of disabilities. Consequently, AG, a spinoff from Space technologies supporting humans in Space, may also support humans who suffer from Gz deprivation on Earth due to lifestyle or medical conditions. Overall, Intermittent AG protocol may effectively “train” cardiovascular hemodynamics or other parameters as neuromotor mechanisms responsible for maintaining orthostatic integrity in young male and female participants with the potential application to space and rehabilitation on Earth.

## Data Availability Statement

The cardiovascular features or the raw data supporting theconclusions of this article will be made available by the corresponding author upon request.

## Ethics Statement

The studies involving human participants were reviewed and approved by the Bioethics Committee of the School of Medicine of the Aristotle University of Thessaloniki, Greece. The patients/participants provided their written informed consent to participate in this study.

## Author Contributions

CK-P, JV, CAF, and PDB conceived and designed the research. CK-P, CN, and CAF conducted the experiments. CAF, CEP., and SG, analyzed the data. CK-P, CAF, CEP, and SG wrote the manuscript. DK, LB, CK-P, JV, and CAF revised the manuscript. PDB and JV supervised the study. All authors contributed to the article and approved the submitted version.

## Conflict of Interest

JV is the director and founder of the ThirdAge company. The remaining authors declare that the research was conducted in the absence of any commercial or financial relationships that could be construed as a potential conflict of interest.

## Footnotes

1 https://www.edwards.com/devices/Hemodynamic-Monitoring/clearsight
