# Supplementary material for: Gravity Threshold and Dose Response Relationships: Health Benefits Using a Short Arm Human Centrifuge
Source: Front Physiol. 2021 May 11;12:644661. doi: 10.3389/fphys.2021.644661 (PMC8144521; doi:10.3389/fphys.2021.644661)
Supplement: Supplementary file 1 [file Data_Sheet_1.PDF]

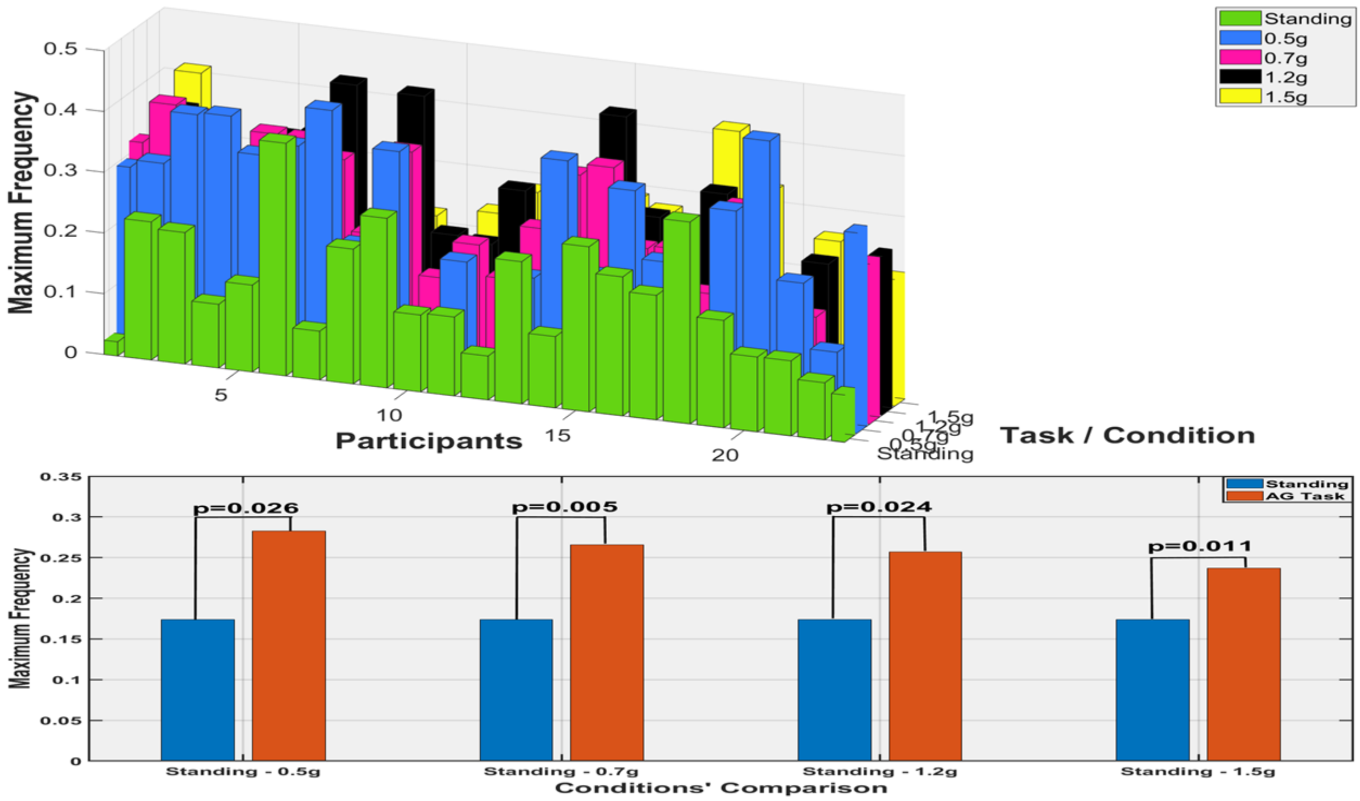

S1: Comparison of maximum Fmax frequency between Standing, 0.5g, 0.7g, 1.2g and 1.5g (upper part). The lower part compares the statistically significant differences in the mean values when Standing is compared to the low and medium intensity G-levels.

S2: Mean values (M) and standard deviations (SD) of the Spectral Properties (Class, Fmax, VLF, LF, HF, VH, LF/HF) in 8 AG levels (standing_5 minutes, 0.5g, 0.7g, 1g, 1.2g, 1.5g, 1.7g & 2g) separately

Spectral Properties	***Property (Mean & Standard Deviation***							
	Standing 5min	0.5g	0.7g	1g	1.2g	1.5g	1.7g	2g
**Class**	2.288 (0.50)	2.880 (0.64)	2.752 (0.51)	2.565 (0.60)	2.710 (0.61)	2.666 (0.43)	2.555 (0.46)	2.434 (0.66)
**Fmax**	0.170 (0.08)	0.288 (0.11)	0.274 (0.09)	0.240 (0.11)	0.265 (0.11)	0.253 (0.08)	0.237 (0.09)	0.207 (0.10)
**VLF**	0.158 (0.11)	0.114 (0.10)	0.120 (0.09)	0.139 (0.11)	0.147 (0.14)	0.132 (0.08)	0.193 (0.15)	0.192 (0.14)
**LF**	0.246 (0.08)	0.200 (0.10)	0.190 (0.08)	0.221 (0.10)	0.203 (0.10)	0.233 (0.12)	0.214 (0.12)	0.290 (0.13)
**HF**	0.438 (0.12)	0.412 (0.10)	0.424 (0.11)	0.405 (0.11)	0.419 (0.13)	0.419 (0.11)	0.393 (0.14)	0.349 (0.15)
**VHF**	0.158 (0.10)	0.274 (0.13)	0.266 (0.13)	0.236 (0.13)	0.231 (0.12)	0.214 (0.13)	0.200 (0.11)	0.161 (0.13)
**LF/HF**	0.648 (0.47)	0.534 (0.34)	0.575 (0.74)	0.687 (0.69)	0.730 (1.01)	0.695 (0.66)	5.013 (21.22)	2.108 (4.18)

S3: Visualization of the main effect of AG levels on LF,VLF,HF,lF/HF and their pairwise comparisons. The results are displayed in terms of their χ^2-, z and p-values. Statistically significant values for LF and LF/HF are highlighted (bold font).

Spectral Property	***χ^2-value***	***p-value***	
**VLF**	13.833	.054	
**LF**	**15.833**	**.027**	
**HF**	5.909	.550	
**LF/HF**	**17.045**	**.017**	
	Pairwise Comparisons	***z-value***	***p-value***
**LF**	0.7g vs 2g	-3.376	.001
**LF/HF**	0.7g vs 2g	-3.406	.001

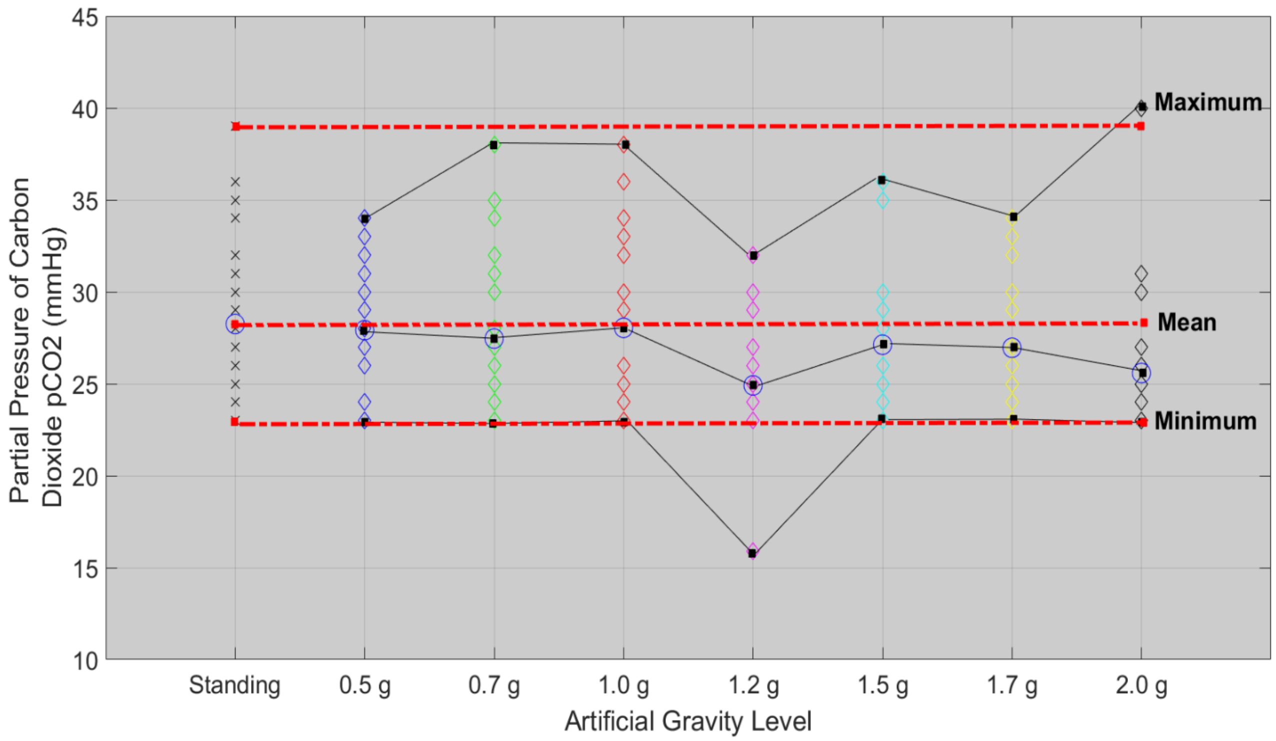

S4: Partial pressure of carbon dioxide for each of the 24 participants in the standing position (red dotted line) and at each Artificial Gravity (AG) level (denoted with a diamond of a different color: blue: 0,5 g, green: 0,7 g, red: 1,0 g, magenta 1,2 g, cyan: 1,5 g, yellow: 1,7 g, black: 2,0 g).

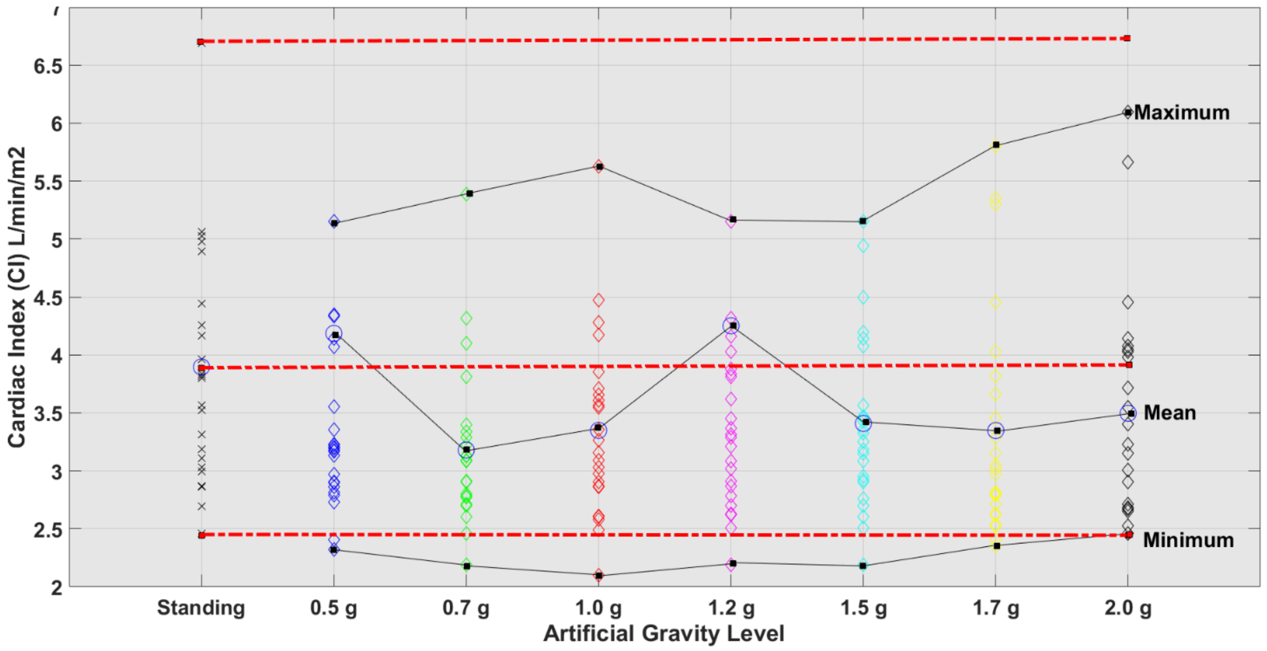

S5: Cardiac Index (CI) for each of the 24 participants in the standing position (red dotted line) and at each Artificial Gravity (AG) level (denoted with a diamond of a different color: blue: 0.5 g, green: 0.7 g, red: 1.0 g, magenta 1,2 g, cyan: 1.5 g, yellow: 1.7 g, black: 2.0 g).

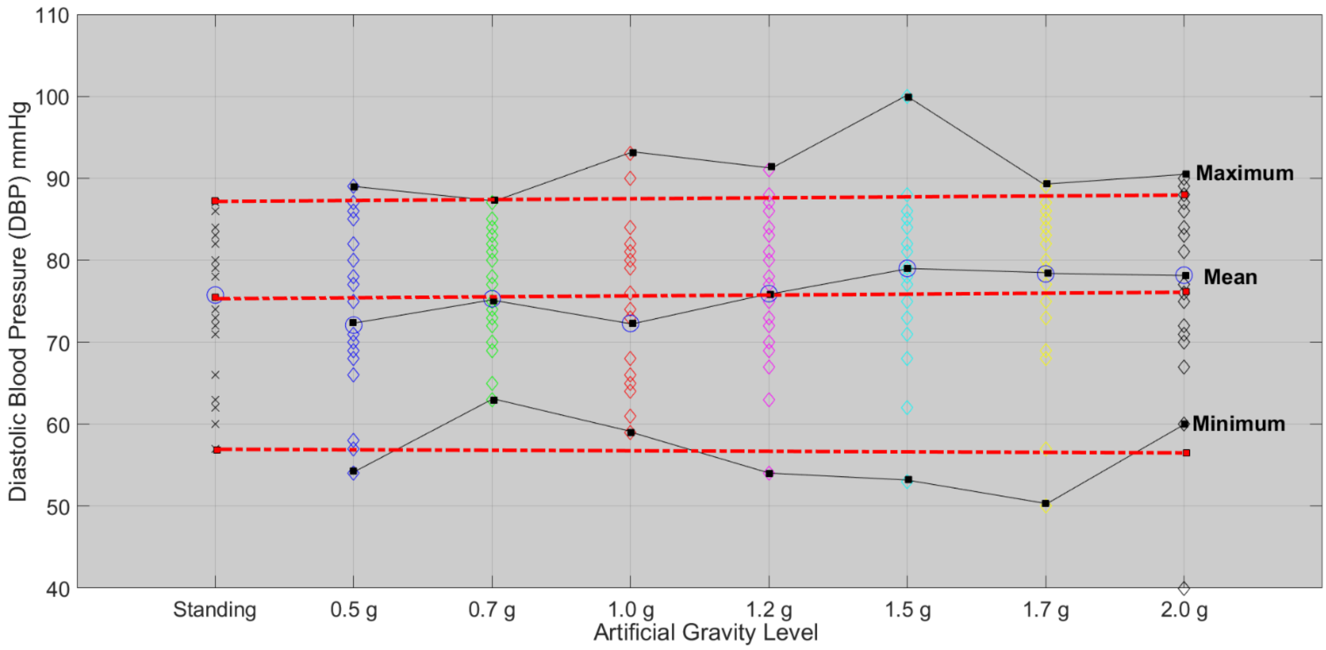

S6: Diastolic Blood pressure for each of the 24 participants in the standing position (red dotted line) and at each Artificial Gravity (AG) level (denoted with a diamond of a different color: blue: 0.5 g, green: 0.7 g, red: 1.0 g, magenta 1.2 g, cyan: 1.5 g, yellow: 1.7 g, black: 2.0 g).

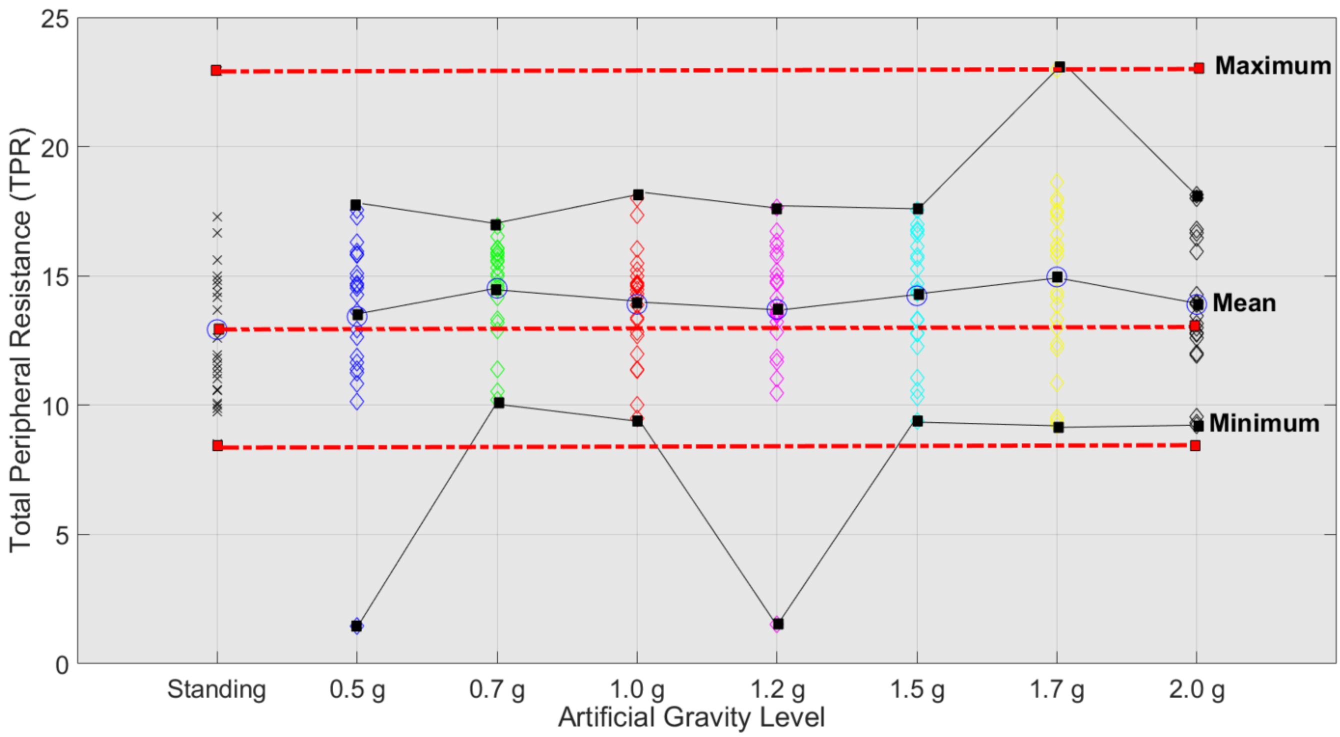

S7: Total Peripheral Resistance for each of the 24 participants in the standing position (red dotted line) and at each Artificial Gravity (AG) level (denoted with a diamond of a different color: blue: 0.5 g, green: 0.7 g, red: 1.0 g, magenta 1.2 g, cyan: 1.5 g, yellow: 1.7 g, black: 2.0 g).
